# Extra Supplement—The Nursing Mirror

**Published:** 1889-06-01

**Authors:** 


### The Nursing Mirror.

All Contributions for this Supplement should be addressed "The Nursing Mirror," care of the Editor, 139-140, Salisbury Court, Fleet Street, London, E.C.

### En Passant.

A NURSE'S PATENT.—A nurse has patented a small portable bath-tub made of rubber cloth. It is put on a wooden framework, light but stout, with pockets on the end for soap, sponge, and so on. The whole shuts up like a camp-stool into a very small space, and is of the greatest convenience in the sick-room or for travelling. Nurses are often so ingenious in their arrangements in a sick-room, that the only wonder is that their inventive faculty has not led some of them to apply more often for patents.

SHORT ITEMS.—Miss Tourtel, matron of the Victoria Hospital, Guernsey, has generously offered to give her services to the hospital without remuneration, which offer the committee has accepted.—The supplement to the June number of *Nursing Notes* consists of an awful list of horrors perpetrated by inexperienced women calling themselves midwives.—At the annual meeting at the Devon and Exeter Hospital on May 23 a strong appeal was made for funds for the proposed nurses' institute.

A NEW MISSION.—The practical spirit which animates modern heroines is shown in the story of Miss Kate Drexel, a New York heiress, which reaches us from America. It was first said that Miss Drexel had entered the Pittsburg Convent; but later and more authentic intelligence states that she has only visited there to receive certain training, and has no intention of becoming a nun. She has, it appears, been for a number of years interested in the Indian and coloured missions, and, not satisfied with giving her money, she wishes now to give herself to this work, and will found a new religious order devoted to the Indians and blacks solely.

REGISTRATION OF MIDWIVES.—A meeting was held at Mrs. Rathbone's last week in aid of the Bill now before Parliament, making compulsory the registration of midwives. Most of the leaders of this movement were present, and it would certainly have astonished the ancient race of Sairey Gamps to see the elegant ladies who were proud to accept the good old English title of midwife, and proclaim themselves holders of the Obstetrical Society's diploma. Mrs. Henry Smith read a paper containing much interesting information, and then Mrs. Scharlieb, M.D., addressed the meeting. Mrs. Scharlieb is the only woman who has yet taken the M.D. degree of London University; she is also physician to the out-patients of the New Hospital for Women, joint Lecturer on Forensic Medicine to the Women's Medical School, etc. For many years Mrs. Scharlieb practised in Madras, and she pointed out that even in India registration is enforced, and that only in England, of all civilised countries, is any woman who likes allowed to put a plate on her door and practise as a midwife without a license. Government protects us from having our teeth drawn by untaught dentists, and protects our dogs and our cows from being attended by unlicensed surgeons; but for our mothers and infants it has taken no care. Mrs. Ormiston Chant next spoke in her usual beautiful voice, which she skilfully used to touch the hearts of her audience. Pathetic, indeed, was the picture she drew of the evil wrought for want of the passing of this Bill. There was some discussion, in which Miss Wilson and Dr. Annie McCall joined, and it was decided that all were to try and stir up members of Parliament to consider this question, and that all were to further it to the utmost of their power. A similar meeting was arranged to be held at Lady Fitzwigrams on Thursday next.

A LADY DOCTOR.—In a late number of the *National Review*, the Hon. N. N. Shore referred to the work done by Miss Trask, M.D., in China. This lady went through a medical college in New York, and then emigrated to Foochow. Her first case was one of dropsy, and she treated it so skilfully that her success amongst the natives was at once assured. Miss Trask has entire control of a hospital for women, and performs surgical operations in numbers. A wide field of usefulness is open to lady doctors in China.

STOKE-UPON-TRENT.—We have received the seventeenth annual report of the Staffordshire Institution for Nurses, and congratulate the committee on the progress it chronicles. During the year 386 cases have been nursed, 15 gratuitously, and 23 on reduced terms. The present staff consists of 58 private nurses, 7 district nurses, and 13 probationers in training. The lady superintendent, Miss Shirley, has a kind word to say about the unselfishness and devotion of her nurses, who are always ready to take any case, no matter what its nature. The home is being enlarged at a cost of £440, the site having been presented by Mr. Bishop. When the nurses of the Stoke Home are ill, their wages are regularly paid; and the committee propose starting a sick fund. We hope they will consider whether it would not be better to affiliate themselves to the National Pension Fund, as so many hospitals and institutions are now doing.

LADY DOCTORS.—When such a note as the following appears in the *St. James's Gazette*, medical women can truly pride themselves on having overcome the public prejudice, which only a few years ago was so bitterly antagonistic to them: "The lady doctors did well to rejoice at the opening of the New Hospital for Women in the Euston-road by the Princess of Wales. The function is one more sign that their order is waxing mighty. They have had much to contend against—neglect, contumely, the discouragement of learned bodies, the jealousy of male rivals, the suspicion of the public. Nevertheless, they persevered—with a quiet energy so different from the shrill vivacity of the shrieking sisterhood of fiction and of fact—and now they have their recognised place, and, it may be added, their recognised rewards. The presence of Rukmabhai, the young Indian lady of whose story the public has heard so much, was an interesting feature at the ceremony. If the female doctors want any justification, there is a single word which justifies them, and that word is India."

DEACONESSES INSTITUTION.—On May 18, the twenty-second annual meeting of the Deaconesses Institution and Hospital, Tottenham, was held, Mr. James Laing, of Sunderland, in the chair. At the present time there are 63 sisters belonging to the institution, who are stationed as follows: At Cork Union, 2; at Sunderland Infirmary, 20; South Dublin Union, 3; King's Cliff Hospital, Scarborough, 2; at the Hospital of the Society for the Propogation of the Gospel among the Jews at Jerusalem, 2; at the Parent Home, Tottenham, 34. The number of patients who had benefited by the attentions of the nursing staff during the year was: at Cork 300, Sunderland 1,731, S. Dublin Union 528, and the Parent Home 801 in-patients, 6,821 out-patients, and dental patients 636, a grand total of 10,819. The chairman bore testimony to the devotion of the sisters, and spoke in high terms of those who were sent down so readily upon request to the infirmary at Sunderland. Sir Edmund Lechemere, M.P., the Rev. G. Turner, Mr. Wilkinson, and others having addressed the meeting, the proceedings were brought to a close by the Benediction. Several of those present stayed to tea with the deaconesses.

### The Healing Touch.

"There *must* be further advice," so said the mild, little country medical man.

He had done what he could for the patient, and with no avail. The vague disease of which she sickened was a mystery beyond his ken. A woman with great wealth—which meant great power and ability to set in motion plenty of schemes whose interests should have sufficed to fill up an empty life—and Mrs. Somers's was so, truly—it seemed a strange thing that out of utter dreariness, the mysterious disease had sprung up in her. A disease that, sapping away her strength of body might ultimately—who could tell?—destroy her mentally also. There was no disguising the fact, the lonely mistress of Somers Court was seriously ill.

"She looks as if she'd slip through our fingers any day," said Simms, her maid, with a tearful sniff.

"So she may," and the little doctor nodded; "that's why I insist on further advice. She must see Sir Hercules. He's *the* authority in such cases. Get her up to town if you can manage it. If any man can bring her round, he's that one."

Somehow—nobody quite knew how—Simms did manage it. She and her mistress had been girls together in the old days, when the bride came to Somers Court, a shy stranger, clinging to her one friend, her maid, the daughter of her own old nurse. The bride's future had not turned out happily—the marriage was a forced one—and when fate made her a widow, she still clung tenaciously to the faithful Simms. Happier days came, and in her little child Mrs. Somers lived over again some of her lost youth. While Geraldine was still a girl, life was very bright, but the girl became a woman, and then the mother's sky darkened. Geraldine made the acquaintance of an adventurer—still worse, a gambler—it was a mad infatuation. Neither mother nor daughter would give way, and it ended in the girl's flight from her old home. Then, Mrs. Somers turned to a woman of stone. She was frozen in her heart; as time went on she grew strangely weak, and as the little doctor said, her case was beyond his skill; he was anxious to shift it on to more competent shoulders.

Sir Hercules was the reverse of the typical pompous physician. He was a little man, all nerves and quicksilver; "Jack-in-the-box," the irreverent students who attended his lectures called him. But Sir Hercules had the true healing-man's gift—the eye that could read off, in the patient's expression, the secret disease. He managed to master that of Mrs. Somers before bowing himself out of the room.

"I want to see one of the family," he said, brusquely, to the butler in the hall.

"There ain't no family, sir."

"Bless me! Nobody? Has she a companion, then?"

"No, sir."

Again Sir Hercules blessed himself. "Well, send her maid. Now, my good woman," eyeing Simms sharply all over, and taking out his watch, "I'm in a hurry, so be as brief as you can. What's on her mind? Out with it! I see you know it."

"Miss Geraldine as was, sir," quavered Simms, who, mind and body, was of the consistency of a jelly, sheer terror of the little great man alone pulling her together.

"Good! That's pat enough. Who's Miss Geraldine, and where is she?"

"The mistress's only child; she ran away and got married. We don't know where she is, sir."

"So! Well, here's my prescription, see it is attended to. Get hold of Miss Geraldine; she's the prescription."

"But, sir—" Simms, all of a tremble, found herself addressing empty air. Sir Hercules, with a hop, skip, and a jump, was down in his carriage, and away into the next street.

"Oh, my patience," sobbed the maid, "how am I to find Miss Geraldine?" Then, like a sensible woman, Simms suddenly dried her tears, and put on her bonnet. She remembered a letter—one of many—that had come from the exiled daughter, and which, unread, had been torn to shreds. As Simms picked up the pieces from the floor she had seen the address on one of the scraps. And weak-headed as she appeared, strange to say, Simms had a memory.

It was a poor, shabby row of houses. Simms, as she entered one of them, shuddered; surely, Miss Geraldine did not live there. In answer to her timid knock, someone called out, "Come in!" In a dull, grimy room sat Miss Geraldine "as was," close to the window, bending over a table strewn with paints, brushes, and pinned-out fans. There was a silence as the two women looked into each other's faces with eyes full—the one pair of dumb anguish, the other of mute pity.

"Oh, Simms! My mother?"

"She needs you sorely, Miss Geraldine. Will you come and save her life?"

There was a slight scuffle on the floor, something tottered across the room, and a golden head, reaching to Geraldine's knee, hid itself in her skirts.

"My patience!" cried Simms, faintly, "what's that?"

"My child, my Gerry; she's all I have in the world, and it is to get her bread that I slave at this work," indicating the gay, flower-painted fans. "Her father is dead," she added in a hard voice. Then, with a bitter cry, she hid her face. "Oh, Simms, I've been terribly punished; he was so bad to her and to me."

"What are you?" Mrs. Somers was gazing, startled, at a fair-haired tiny mortal, whose blue eyes returned her stare with interest.

"I is Gerry," spoke up a small, sweet voice.

Mrs. Somers gasped; then she prayed fervently to be delivered from the madness she felt had stricken her, for madness it must be to imagine that the clock of time had put back its hands long years to bring her child, her Gerry, back once more.

"Is you frightened?" gravely asked the little voice, and its owner staggered close up to the couch. Soft baby fingers crept about the stony face, and at their touch Mrs. Somers sat suddenly upright, wildly clasping the tiny creature to her. The frozen heart melted, and there was a torrent of scalding tears.

"Don't ky," murmured small Gerry, but when her shrinking mother, followed by faithful Simms, stole into the room, they knew those tears were the patient's salvation. And it came to pass that the other Geraldine, forgiven, was taken also into the mother's arms empty so long.

The little doctor was jubilant when his patient returned home to Somers Court—cured!

"I told you Sir Hercules was the man! But where's his prescription? I should like to see it," he asked inquisitively.

"Then you shall, doctor. See, here, it is, my Geraldine!"

### Homes in the Hills.

The following is an extract from a letter from Lady Roberts, which has lately reached England:—"I wish I could show you the Nurses' Home and Officers' Hospital in connection with it which I have been enabled to establish at Murree. The whole appearance of the Soldiers' Hospital is also changed since the nurses took charge of it, and the men look so happy and contented that it is quite a pleasure to go and see them. Subscriptions, I am sorry to say, are now coming in very slowly to my fund, and I fear I shall have very great difficulty in keeping the work going." Lieutenant-Colonel Gildea adds: "No appeal has ever yet come from India to England without meeting with a hearty and liberal response, and no appeal could more come home to the people of all classes in this country than one which provides for the health of the ladies who are devoting their lives to tending and nursing those who have made the Indian Empire the brightest jewel in the crown of England. There should be little or no difficulty in providing Lady Roberts with at least five or six thousand pounds, to enable her to place 'her homes' at once on a more permanent footing. Subscriptions, which will be duly acknowledged in the daily papers, may be paid to Her Royal Highness the Princess Christian's Fund, through Captain Wilford Lloyd, Naval and Military Club, Piccadilly; to any office bearers of the Soldiers' and Sailors' Families' Association throughout the country; to Col. Gildea himself as treasurer; or to the account of Lady Roberts' 'Homes in the Hills,' at Messrs. Coutts and Co."

### A Wander Round Guy's.

THERE is a delightful old world flavour about some of our hospitals, such as Bart's, the London, and Guy's. We look at the well-worn stairs and the balusters black with age, and we think of the many ghosts who must surround us if "ghost walking" is a fact; why, the air must be thick with them. Even as one gets to love Merton or Magdalen College, do you get to love the dusty passages and quiet wards of a hospital where you have been a student. Every alteration and improvement is regarded with disgust, and while acknowledging that medical science ought to advance, the introduction of ventilators into the venerable walls is looked on as a desecration. On revisiting Guy's quite lately, the numerous improvements I saw made me quite sad; old landmarks were gone, old traditions forgotten, and I sought in vain to find something "just the same" as it had been in my days. The least change was in the wards, but even here the dear old blue check curtains had given place to flaunting pink things, and though the sisters forced me to confess that the pink was brighter and prettier, I longed for the homely blue check. Then in one ward I saw a semi-circular sort of stand for teaching paralytics to walk; why, in my day a nurse supported the patient on one side and perhaps a student supported him on the other! "This machine is much more convenient," said the stern sister. Yes, but I hated the look of the mahogany thing. I must say the neat little tables containing all materials for putting on a plaster of Paris splint struck me as useful, and the sisters' new baskets were neat and handy. I made my way to the pay wards, which were quite new to me; two long wards divided into cubicles, and brightly fitted up, one ward for women and one for men. Both wards were quite full, and the sister expected to shortly open other two wards which were in readiness, but had not yet been used. These paying patients have a medical officer entirely to themselves, and are well taken care of. If ever I have to have an operation performed I think I shall go to Guy's, and try what it is like to be a patient in those familiar precincts.

Leaving the wards, I made my way to the dispensary, where medicine is made by the gallon, and is delivered at the door in barrels, like beer. An apparatus for making nitrous oxide gas was the newest thing here—and a picture of a pretty woman in the dispenser's private room! In the kitchen things were much the same; huge vats of beef tea, ovens which were like small chambers, and whole flocks of sheep roasting within them. No—there were some innovations; a machine for cutting bread, for instance; and I heard of a plan for giving all the patients fish once a week, which has been successfully done at Bartholomew's. From the kitchen into the nurses' dining-room is just a step. The dining-room is underground and lit by skylights; it used to be a dingy place, but halfway up the wall at the top end of the room is the bright frescoe of Spring, lately given by the Academy. The coloring is soft, yet clear, and a more cheerful picture for tired nurses to gaze on could not be imagined.

From there I made my way to the museum, for no one can ever weary of studying the exquisite wax models of every part of the human body for which Guy's is famous. What wouldn't you nurses give for a peep at them? How quiet the long narrow room is! There is a student absorbed in studying the muscles of the hand and arm; he does not hear me as I approach, but just as I get behind him he moves suddenly and his face crimsons—he shuts a knife with a snap and moves away; instead of gazing on the wax model of a dissected human arm he has been carving a woman's name on the wooden table! Ah, well, I did the same myself once; Guy's may change, but human nature never changes.

### Death in Our Ranks.

THE funeral of the late Miss Bessie Hunt, charge nurse of No. 6 Ward, Bristol Royal Infirmary, took place at Arno's-vale Cemetery, on May 23, the Rev. Fairfax Goodall officiating. There were present, together with personal friends, Miss Gibbs, Miss Stephenson, Dr. Waldo, Mr. J. Paul Bush, Mr. John Dacre, the resident medical officers, the matrons, and many nurses of the infirmary. Several beautiful wreaths were sent. Nurse Hunt had faithfully performed her duties for fourteen years. Her exemplary conduct, amiable disposition, and tender kindness to all under her care caused her to be universally regarded with the highest esteem.

### Everybody's Opinion.

[*Correspondence on all subjects is invited, but we cannot in any way be responsible for the opinions expressed by our correspondents. No communications can be entertained if the name and address of the correspondent is not given, or unless one side of the paper only be written on.*]

### THE BRITISH NURSES' ASSOCIATION.

A MEMBER OF THE B.N.A. writes: Allow me to point out one or two mistakes made by a "Hospital Certificated Nurse" in THE HOSPITAL of May 18. In quoting from the pamphlet published by the British Nurses' Association she gives this sentence: "The British Nurses' Association guarantees that the person to whom this word 'nurse' is applied has passed through a prescribed course of study and practice." The actual words are: "At present, when we hear that a woman is a nurse, we hardly know what meaning to attach to the word; but when the association has succeeded in the attainment of its primary object the meaning will be no longer doubtful: *it will be* (the italics are my own) that the person to whom the word is applied has passed through a prescribed course of study and practice," etc. The "primary object" of the British Nurses' Association is to obtain "a Royal charter, which will authorise the registration of nurses, on terms satisfactory to physicians and surgeons, as evidence of their having received systematic training." The association does not pretend now to test the nursing abilities of its members—it has no power to do so—but it hopes it may some day see nursing "an acknowledged and legally constituted profession," whose members are "united together for all legitimate purposes of mutual protection and help." The "magic certificate" is simply a card sent to a nurse to inform her that she has been enrolled as a member. I believe it to be no unusual thing for an association to have some badge or sign of membership. I have failed to find the words, "know what meaning to attach to the words 'member of the British Nurses' Association,'" in the pamphlet, which can be obtained from the Secretary, 8, Oxford-circus-avenue, W.

TRUTH writes: In the begging circular just issued by the British Nurses' Association, the council aims at obtaining from the public a yearly income equivalent to a capital of over £400,000 at three per cent. We are told that this trifling sum is required "to protect the public from the hundreds of unskilled workers who now, without let or hindrance, pretend to act as trained nurses, and by their ignorance cause incalculable suffering and danger to the sick." Now, what nonsense this is. Is it not insulting the common-sense of the public to say that its sick members cannot, with the exercise of moderate care, obtain a thoroughly reliable nurse from one of the many responsible homes throughout the country? Is is not insulting the medical profession to urge that any medical man could be so blind to the condition and surroundings of his patient as not to perceive when a nurse is causing an amount of "suffering and danger" so great as to defy calculation? No, sir, it is not the public whom the promoters of this scheme seek primarily to benefit, but themselves. Why, then, should the public pay for it? In conclusion, I beg to enter my protest as a medical man against the iniquitous system of encouraging nurses to beg from their patients, and to stigmatise it as humiliating and unprofessional.

### MASSAGE.

Miss Clark writes: In this week's Hospital ("Notes and Queries," No. 18) "Leamington" asks: "What is the best book on massage?" So far, I think, Dr. Douglas Trahern's "Practical Treatise of the Art and Practice of Massage" (New York: Woods and Co.) is undoubtedly the best. It can be obtained at Lewis's, 136, Gower-street. This month, however, Dr. Spetch Dowse, of Welbeck-street, has published a book on the subject. It is now nearly three years since I took up the subject of electro-massage, and, holding certificates from the West End Hospital, and also Dr. Dowse's private class, I believe I am experienced from an English point of view, but I consider the manner in which certificates are given to women after six or a dozen lessons is simply ruinous to the whole subject. Every nurse or rubber who has bought a battery calls herself an electro-masseuse, and creates prejudice among ordinary practitioners against a very valuable method of treatment. I would thankfully give up my own certificates if it were rendered illegal for any but a qualified medical man or woman to apply electricity.

### THE FOOD QUESTION.

Twenty-five Nurses write: In The Hospital of May 4 a paragraph on "The Food Question" has attracted our attention. So far as this hospital (North-Western, Haverstock-hill,) is concerned, we desire to state that the rations supplied are abundant in quantity, of good quality, varied as far as opportunities occur, and, upon the whole, well served. It seems only fair to the authorities of this institution to make this statement.

### HOMES OF REST.

F. D., writes: Seeing the enquiry of a governess for a home of rest, and that of a nurse for a convalescent home, I enclose you the pamphlet of the Merchant Taylors' Home at Bognor. You may, of course, know of it; but I do not think it is generally known among women-workers that there is a home of that class entirely free, and where travelling expenses (from and to London) are paid. It has been my privilege to be received as a guest at the Bognor Home, and I gratefully say that the month I spent there last year was one of the happiest I remember. The patients are chiefly governesses, lady clerks, students, and some nurses—more of the last, I believe, would apply, if they knew of this institution. I should say it is just the place for a poor fagged nurse. The house is replete with every comfort, and its inmates are entirely free to go out or stay in as it pleases them, or to keep in their own rooms if not strong enough to bear companionship. During the summer the patients often number as many as forty, but one does not feel at all overwhelmed by the number, as besides large drawing and dining-rooms there are three other sitting-rooms, and a nice library for the use of patients at all times. The diet is generous, and appointments refined, and the place altogether is quite unlike an institution, but is really in every respect a home. There is another free home at Worthing, "The Thomas Banting Memorial," which I have heard spoken of very highly. Twelve ladies are received there entirely free of charge. Patients pay their own travelling expenses, but are provided with a pass which enables them to travel second class at third-class rate. I think there is more delay in gaining admission to this home than to Bognor on account of the number received being so much smaller, but, of course, those who know just when they will have their holidays could always apply a week or two before. For particulars of this home applicants must apply to the Secretary, Thomas Banting Memorial Home, Parade Lodge, Marine-parade, Worthing. I read The Hospital with much interest. I am not, as yet, a nurse, but am shortly going to begin training for one, and I feel it a great advantage to hear both sides of the nursing question beforehand.

### NURSES OR WATER-NYMPHS?

Allopathy writes: I would like to call attention to a subject, which in almost all cases proves a source of annoyance to the parties concerned. I refer to the sending of allopathy nurses to wait upon homœpathy or hydropathy cases, and, as an instance, I may cite the following, which is by no means an exceptional one. I was sent out lately to attend upon a case of advanced phthisis. The patient had spent two years in hydropathic establishments, and, eventually, getting worse, she was sent home to die. On arriving, I presented the rules of our institution, which require the nurse to take her instructions from the medical men in attendance. A doctor was called in next day, and asked to visit occasionally, but to what purpose? Merely to be scoffed at behind his back, and have his suggestions thrown ignominiously aside; the customary head washing, feet washing and cold-water cloths taking their place. Now, this is very unpleasant, and in order to remedy the state of matters existing, might I suggest, that hydropathic establishments keep a few trained water-nymphs, or bathmaids (whatever they like to call them), who are capable of waiting upon patients in such cases?

### THE AGE OF NURSES.

J. G., Matron, writes: Will you kindly allow me to endorse Nurse M. J.'s sentiments concerning the age of nurses. There has been of late years such a marked tendency to place young people in positions of authority—with what success, it is not for me to say. It is the case with matrons and sisters as well as with nurses. In applying for the matronship of an hospital, the candidates over forty are entirely put on one side for younger and far less experienced ones. Therefore, if a matron, sister, or nurse is out of work at 45, what is to happen to her till she comes in for her pension at 60? She is not ill, and cannot claim sick pay; but she is to be put on one side for younger people.

### Appointments.

[It is requested that successful candidates will send a copy of their applications and testimonials, with date of election, to The Editor, The Lodge, Porchester-square, W.]

Salford District Nurses' Home.—Miss C. E. Barff has been unanimously elected matron of this home. Miss Barff trained at the Nightingale School, St. Thomas's, in 1881, and was for some time sister of a medical ward there. Afterwards Miss Barff learnt district work at the National and Metropolitan Training School, whose centre is in Bloomsbury-square. From there she was appointed matron of the District Nurses' Home, Liverpool. We feel sure many readers will be glad to hear of this appointment, as Miss Barff has taken temporary duties in many places, and has a wide circle of friends.

Bradford Infirmary. — Mrs. F. Magill, formerly assistant matron at Liverpool Royal Infirmary, has been appointed lady superintendent at Bradford.

### Vacancies.

The following vacancies are announced:

**Matrons.**

Bournemouth Consumption Sanatorium, £50.

Beckenham Cottage Hospital, £40.

Gartnavel Asylum for Lunatics, £80.

King's Cross Hospital, Dundee, £70.

**Nurses.**

Albert Hospital, Devonport, £25.

Barton-on-Irwell, Manchester.

Belgrave Hospital for Children.

Bolton District Nurses' Home, £25.

Bradford Fever Hospital (two), £23.

Carmarthen Infirmary, £23.

Eastbourne Nursing Institution.

Glamorgan Infirmary.

Heart Hospital, Soho-square.

Hull Nursing Institution, £25.

Kingston Home, Hull.

Liverpool Convalescent Institution.

Milton Fever Hospital, Portsmouth (assistant), £16.

Nottingham Nursing Institution.

Nurses' Institute, Bedford.

Providence Home, Nottingham.

Rochdale Infirmary, £22.

Stockport Infirmary (two), £24.

Stratford-on-Avon Hospital, £20.

St. Cuthbert's Poorhouse Hospital, Edinburgh (two), £25.

St. George's Home, Sheffield (two).

Wimbledon Nursing Institution, Fitzroy House.

Wirral Children's Hospital, Birkenhead, £22.

Workhouse Infirmary Nursing Association (several).

**District Nurses.**

Burton-on-Trent Institution, £26.
Hersham, Walton-on-Thames, £26.
Salford District Nurses' Home, £25.

**Probationers.**

Bath Nurses' Home.

Burton-on-Trent Institution.

Macclesfield Infirmary.

Stoke-on-Trent Home.

### Examination Questions.

(The Editor will be glad of specimens of similar questions.

### SENIOR MEDICAL.

(1) A patient suddenly vomits blood, what ought a nurse to do?
(2) How would you air and sweep a room with a patient sleeping in it?
(3) How do you make beef-tea, linseed-tea, and mutton-broth? How do you peptonise food?
(4) What are the chief substances used in Enemata, and how are they prepared?
(5) What points does a nurse watch in a case of Enteric Fever?

### SENIOR SURGICAL.

(1) What are nature's means for regulating the temperature of the body?
(2) What should the length, breadth, and height be of a ward for 16 patients?
(3) How should you prepare a patient for Ovariotomy?
(4) How should a tent be prepared for Tracheotomy case? What precautions should a nurse take if it is a case of Diphtheria?
(5) What is the cause of Ringworm? How should a nurse apply ointment ordered for such cases?

### Notes and Queries.

**Queries.**

(22) Will a reader kindly tell me what the action of quinine and antipyrin is on the uterus? *A. P.*

**Answers.**

(14) *Home of Rest.*—Apply to Finsbury House, Ramsgate.

(18) *Massage.*—Dr. Douglas Trahern's "Practical Treatise on the Art and Practice of Massage"; New York, Woods and Co. *A. G. C.*

(16) *Microscope Glass.*—The Manchester Microscopical Society might suit *W.* Last week's meeting was devoted to practical work in the mounting section, Mr. W. H. Tyas in the chair. *Student.*

